# Antioxidant Capacity of Tempura Deep-Fried Products Prepared Using Barley, Buckwheat, and Job’s Tears Flours

**DOI:** 10.3390/foods9091246

**Published:** 2020-09-07

**Authors:** Asuka Taniguchi, Nami Kyogoku, Hiroko Kimura, Tsubasa Kondo, Keiko Nagao, Rie Kobayashi

**Affiliations:** 1 Graduate School of Humanities and Life Sciences, Tokyo Kasei University, Tokyo 1738602, Japan; g190301@tokyo-kasei.ac.jp (A.T.); kyogoku@kanazawa-gu.ac.jp (N.K.); nagao@tokyo-kasei.ac.jp (K.N.); 2 Department of Food and Nutrition, Kanazawa Gakuin College, Ishikawa 9201392, Japan; 3 Faculty of Home Economics, Tokyo Kasei University, Tokyo 1738602, Japan; 716hiroko.w@gmail.com (H.K.); kondo-t@tokyo-kasei.ac.jp (T.K.)

**Keywords:** tempura, deep-fried product, barley, buckwheat, Job’s tears, antioxidant capacity, oil deterioration, polyphenol

## Abstract

Tempura is a dish of battered and deep-fried foods, and wheat flour is typically used; however, barley, buckwheat, and Job’s tears have an antioxidant capacity. This study investigated whether replacing wheat flour with flours from these three crops in tempura affects the antioxidant capacity and deterioration of frying oil. Radical scavenging activity and polyphenol content of tempura were measured by chemiluminescence-based assay and the Folin–Denis method, respectively. The peroxide value, *p*-anisidin value, acid value, and polar compound of the oil used in frying were measured as indexes of oil deterioration post-frying due to oxidation. Although the frying oil of barley showed higher *p*-anisidin value than that of wheat, the oil samples’ deterioration level measured in this study was low. The antioxidant capacity and polyphenol content in the three flours samples were higher than those in wheat sample, with buckwheat producing the greatest values, followed by Job’s tears, and then barley. Thus, deep-fried products prepared using the three flours demonstrated superior antioxidant capacity owing to the abundance of antioxidant components. Therefore, tempura can be enjoyed in a healthier manner by using batter prepared using those flours, and substituting wheat flour with the three flours can increase the antioxidant capacity of deep-fried products.

## 1. Introduction

Tempura is a traditional Japanese dish that has gained popularity all over the world. It is prepared from a batter of water and flour; sea food or vegetable is lightly dipped in the batter and then instantly fried [1]. In general, wheat flour is used to prepare tempura; however, the structural characteristics of gluten present in wheat flour disturb the process of cooking high-quality tempura. The quality improvement of tempura has been studied: Nakamura et al. [2] used rice flour in their experiment; Matsunaga et al. [1] added different types of starch from various plants to wheat proteins, and Carvalho et al. [3] replaced water during batter preparation with ethanol and a CO_2_ injection. Each of the methods described above effectively improved the quality of tempura. Another study by Martínez-Pineda et al. [4] prepared tempura using batter with malt dextrin, ethanol, baking powder, and corn flour. They concluded that adapting batter ingredients according to the purpose was an effective strategy to improve the quality of deep-fried products. Despite these studies conducted on tempura quality, no research has examined the use of barley, buckwheat, and Job’s tears that do not construct gluten structure in preparing tempura.

In addition, these flours have several health benefits [5,6,7]. Regular intake of these grains reduces the risk of several chronic diseases associated with oxidative stress. Therefore, the biofunctionality of these flours is an attractive option for people who are looking for healthier options. Studies have demonstrated that these grains have high antioxidant capacities [8,9]. However, few studies have focused on the antioxidant capacity of these flours in deep-fried products, especially tempura.

We hypothesized that these flours of barley, buckwheat, and Job’s tears not only provide health benefits but also make cooking tempura easier and improve its texture. We hope that research on tempura prepared using these flours will help establish their wider use for these flours. The quality and palatability of tempura prepared using those flours have already been evaluated in our previous study [10]. Hence, this study examined the antioxidant capacity of tempura prepared using barley, buckwheat, and Job’s tears flours.

In the human body, the antioxidant activity of foods sequesters reactive oxygen is generated by electron reduction during metabolism [11]. Usually, reactive oxygen acts on immune functions [12], but when these oxygen groups are over produced, they cause lifestyle-related disease. Although the human body has antioxidant enzymes to extinguish reactive oxygen [12], they are not enough to inhibit excess active oxygen. The peroxyl radical, which is secondarily generated from the oxidation of cell lipids by active oxygen generated with energy production, is a particularly toxic reactive oxygen species, which promotes serial oxidation and spreads cell disorder. To erase peroxyl radicals, which cannot be absorbed or sequestered by other means in the human body, antioxidant activity in foods is essential. Antioxidant components derived from foods for peroxyl radicals are expected to reduce the risks of lifestyle-related diseases.

In addition, it is necessary to manage the quality of oil as tempura is a dish in which the ingredients are deep-fried in oil. In Japan, there are standards [13] for foods that use a lot of oil during cooking to manage oil quality. One of the rules has to do with peroxide value (PV) and acid value (AV). These are indexes of fat deterioration in foods. Oil deterioration causes a change in smell, taste, and color and decreases nutritive value. Deteriorated oil gives detrimental effects, such as nausea, abdominal pain, and lassitude to the human body. Thus, deterioration of oil is an important index and is related to health function. Since the deterioration mechanism of heated oil is complicated and its evaluation is necessary using multiple indicators, *p*-anisidin value (AnV) and polar compound (PC) were also examined.

Therefore, the present study investigated whether the use of three flours of barley, buckwheat, and Job’s tears in preparing tempura could be useful for increased antioxidant capacity as a new added value. In this analysis, we evaluated the antioxidant capacity of their tempura based on peroxyl radical scavenging activity, polyphenol content, and oil oxidation.

## 2. Materials and Methods

### 2.1. Ingredients

Barley flour (Fiber snow; six-row barley, JA Komatsushi), buckwheat flour (common buckwheat, Nikkoku Seifun Corp., Nagano, Japan), and Job’s tears flour (Akishizuku, Takada Hiryo–ten Co., Tochigi, Japan) were used. In addition to these flours, weak wheat flour (Hoshihime, protein content 8.8 g, Nikkoku Seifun Corp., Nagano, Japan) was used to compare with the three flours.

### 2.2. Preparation of Tempura Samples

The water content ratio for tempura is usually 160% of the weight of the wheat flour. Water ratios for the barley, buckwheat, and Job’s tears flours were adjusted until the viscosity based on the steady flow measured using a rheometer (HAAKE MARS II, Thermo Fisher Scientific, Inc., Waltham, MA, USA) of the resulting batters was consistent with that of the wheat-based batter [14]. These ratios were determined to be as follows: barley, 260%; buckwheat, 190%; Job’s tears, 190%. After adding water to each flour, they were stirred 60 times at a speed of once every second. The buckwheat and Job’s tears batter were left to rest for 30 min, and then they were stirred again 10 times. The four batters were dispensed into silicon cups (*φ*30 mm, height 10 mm, Daiso Industries Co., Ltd., Hiroshima, Japan) by 2.0 mL each and fried in canola oil (stored unopened in a dark and cool place, The Nisshin OilliO Group, Ltd., Tokyo, Japan) for 140 s at a temperature of 180 °C–190 °C using a fryer (CDF-100, Cuisinart Compact Deep Fryer, Cuisinart, Stamford, CT, USA). After cooling for 1 min, they were preserved in a freezer (MDF-300E1, deep freezer, Fukushima Industries Corp., Osaka, Japan) set at −80 °C for at least 24 h. In the following measurements, six tempura samples were milled and analyzed in each test.

### 2.3. Preparation of Oil after Frying Samples

**Figure 1:**
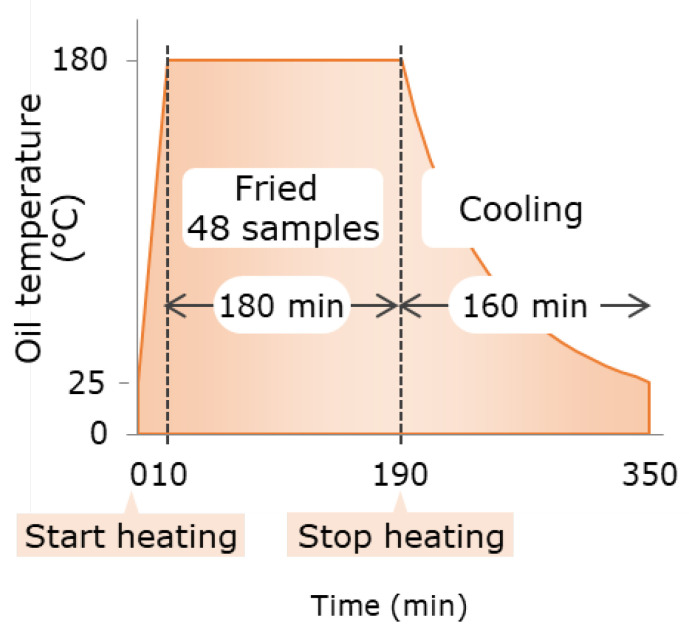
Tempura frying process used in this study.

After frying, the oil was used for PV, AV, AnV and PC analysis. Frying oil samples were heated for 3 h at 180 °C ± 5 °C. During this time, 48 samples of tempura were fried and allowed to radiate heat naturally after heating (Figure 1).

### 2.4. Chemiluminescence Method

After preliminary freezing, the samples were freeze-dried (DC800, Freeze Dryer, Yamato Scientific Co., Ltd., Tokyo, Japan) and then crushed using a mill (IFM-800, Iwatani Corp., Osaka, Japan). Crushed samples were used for analysis of chemiluminescence and measurement of peroxyl radical scavenging activity, as described previously [15]. The results were measured using a photon counter (Lumitester C-100, Kikkoman Co., Ltd., Chiba, Japan) and shown as IC_50_ values. IC_50_ was defined as the sample density that was half the light emission value of the phosphate buffer solution without an antioxidant component. This was calculated as previously reported [15], and the results were expressed in Trolox equivalents.

### 2.5. Analysis of Oil Oxidation

Frying oils include various components, such as peroxides, which are produced by primary oxidation; carbonyl compounds, which are secondary products of this oxidation; free fatty acids, which are produced by hydrolysis, and polymerized glycerols. In the present study, PV, AV, AnV, and PC were measured to evaluate the complicated deterioration of frying oils. The AV, AnV, and PC were measured according to the standard methods of the Japan Oil Chemists’ Society [16].

PV was measured based on previous standards [13]. Sample oil (5 g) was mixed with 10 mL of chloroform (FUJIFILM Wako Pure Chemical Corp., Tokyo, Japan) and 15 mL of glacial acetic acid. Potassium iodide saturated solution (1 mL; FUJIFILM Wako Pure Chemical Corp.) was added, which was prepared with 70 g of potassium iodide dissolved (FUJIFILM Wako Pure Chemical Corp.) in 50 mL of boiled deionized water and shaken intensely for 1 min. They were left for 10 min in the dark, and after the addition of 75 mL of deionized water, the samples were again intensely shaken. Subsequently, they underwent titrimetric analysis with 0.01 M sodium thiosulfate (FUJIFILM Wako Pure Chemical Corp.), which is a standard solution, and 1% starch solution, which is an indicator that 1 g of soluble starch (FUJIFILM Wako Pure Chemical Corp.) was boiled and dissolved with 100 mL of deionized water. The 0.01 M sodium thiosulfate was diluted from 0.1 M sodium thiosulfate that had been previously quantified. This titration comprised the blank containing no oil. The formula to calculate PV was as follows:

PV = 0.01 × (A – B) × F × 1000/S(1)

where A is the titration amount of 0.01 M sodium thiosulfate in the main examination (mL), B is the titration amount of 0.01 M sodium thiosulfate in the blank examination (mL), F is the titer of 0.1 M sodium thiosulfate, and S is the weight of the oil samples (g).

### 2.6. Polyphenol Contents

Total polyphenol content in each flour type and tempura sample were measured by the Folin–Denis method [17]. Each flour type or sample (1.0 g) was mixed with 80% ethanol/water solution and heated to 80 °C while stirring with a magnetic stirrer. After 30 min, they were centrifuged at 3000 ×*g* for 10 min and then treated as extracted samples. A 1.0 mL aliquot from each extracted sample was mixed with 1.0 mL of Folin & Ciocalteu’s Phenol reagent (MP Biomedicals, Inc., Santa Ana, USA) and left for 3 min. Sodium bicarbonate solution (1.0 mL, 10% w/w) was added, stirred, and reacted for 60 min at a room temperature. The absorbance at 700 nm was measured using a spectrophotometer (UH5300, HITACHI Ltd., Tokyo, Japan). Results were expressed as mg of catechin per 100 g of sample.

### 2.7. Statistical Analysis

Means ± standard deviation was calculated and analyzed for significant differences by Tukey’s test of multiple comparisons after performing analysis of variance using R software (3.3.4). For samples, values of *p* < 0.05 were considered significant.

## 3. Results

### 3.1. Antioxidant Capacity of Barley, Buckwheat, and Job’s Tears Flours and Their Tempura

**Figure 2:**
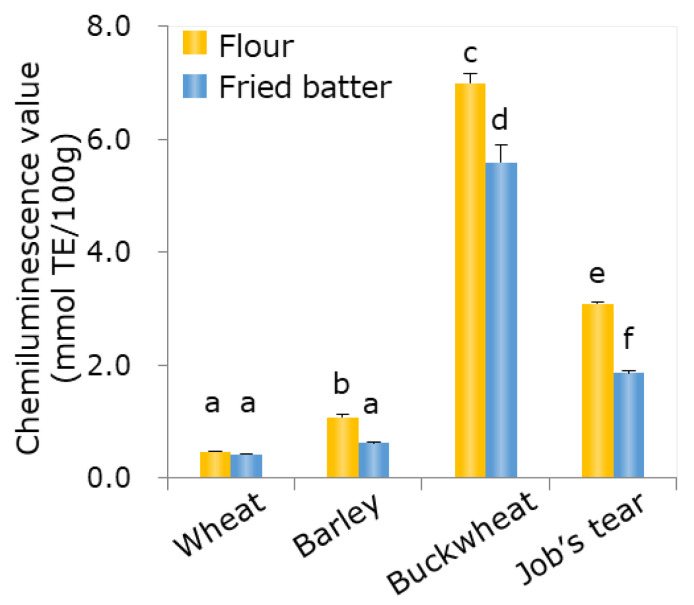
Peroxyl radical scavenging capacity in the four flours examined in this study. The IC_50_ value was defined as the sample density that was half the value of the phosphate buffer solution wherein light emissions did not include an antioxidant component. Results are expressed as Trolox equivalents and measured using a chemiluminescence-based assay. Each value represents the mean ± SD (*n* = 3). Different letters a-f for flours and fried batter indicate a significant difference (*p* < 0.05, Tukey test).

Peroxyl radical scavenging activity was measured and expressed in Trolox equivalents. In this unit, a higher value indicates a higher antioxidant capacity (Figure 2). The antioxidant values of the flours of barley, buckwheat, and Job’s tears were significantly higher than those of wheat flour. When comparing the flour and the fried batter in barley, buckwheat, and Job’s tears, the antioxidant values of fried batter were significantly lower than that of the flour. However, the antioxidant values of buckwheat and the Job’s tears samples were higher than that in the wheat sample also in fried batter. Although the average value of the radical scavenging activity was higher in the barley sample than in the wheat sample, there was no significant difference between the barley and wheat samples. 

### 3.2. Oxidation of the Oil Used in Frying

In this study, PV, AV, AnV, and PC were measured to evaluate the oxidation of the oil used for frying. 

AV is the concentration free fatty acids in the oil due to the hydrolysis of oil [18], and PC measures oxidization, dimerization, and polymerization of triacylglycerols and diacylglycerols and free fatty acids [19]. AV was 0.1 for all sample oils, while PC was as follows: wheat, 6.0% ± 0.24%; barley, 5.7% ± 0.42%; buckwheat, 5.4% ± 0.12%; Job’s tears, 5.6% ± 0.26%. These indexes of frying oils were at the same level, and there was no significant difference. 

PV and AnV were compared, and the results are showed in Table 1. PV is an index of peroxides; however, since peroxides are thermally unstable and decompose at high temperatures [20], AnV that evaluates its decomposition products, aldehydes, and ketones was examined. The oil samples used for frying barley, buckwheat, and Job’s tears flours had a lower PV than that used for wheat flour. Although the oil used for buckwheat and Job’s tears samples did not show significant difference compared with the oil used for wheat and barley sample, AnV of the oil used for barley sample was higher than that used for wheat sample.

### 3.3. Polyphenol Content

**Figure 3:**
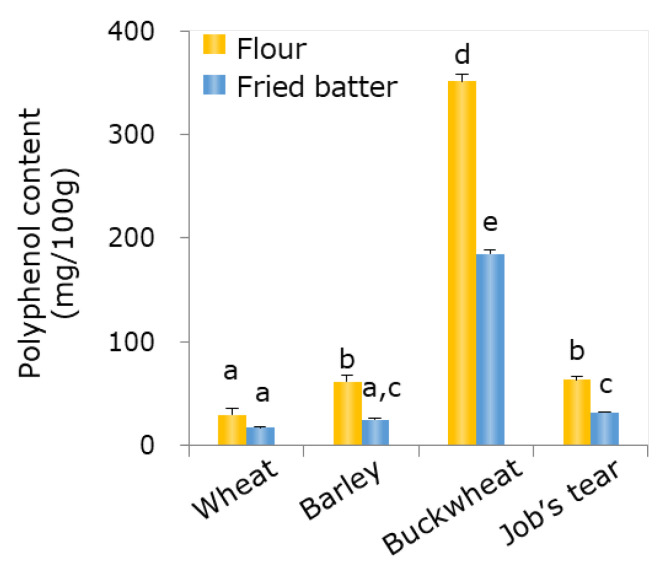
Polyphenol content in each of the flours examined in this study, measured using the Folin–Denis method. Each value represents the mean ± SD (*n* = 3). Different letters a-e for flours and fried batter indicate a significant difference (*p* < 0.05, Tukey test).

As with antioxidant capacity, barley, buckwheat, and Job’s tears flours displayed higher polyphenol content than wheat flour (Figure 3). Although the polyphenol content was significantly decreased by frying, tempura samples prepared using buckwheat and Job’s tears flours maintained high levels of polyphenol. The polyphenol content of barley sample was between that of wheat and Job’s tears samples, with no significant difference. The polyphenol content in tempura was highest for buckwheat, followed by Job’s tears, barley, and finally wheat. This was the same order as that of antioxidant capacity.

## 4. Discussion

In this study, we investigated whether substituting wheat flour with flours of barley, buckwheat, and Job’s tears in deep-fried batter enhances tempura’s antioxidant capacity and suppresses deterioration of the frying oil. In addition, we also examined the contribution of polyphenol present in those grains to these effects.

It was confirmed that the peroxyl radical scavenging activity in barley, buckwheat, and Job’s tears flours was significantly higher than that of wheat flour. Among the three flours, buckwheat and Job’s tears samples had high peroxyl radical scavenging activity, even after deep-frying. In particular, the buckwheat sample had the highest antioxidant value among the four flours examined, comparable with that of ginger [21], which is known to possess antioxidant activity [22]. Although other studies have reported that the use of these flours in baked products increases the antioxidant capacity [23,24], this study found that similar effects could be achieved with deep-fried products using these flours.

In the analysis of the deterioration process of frying oil, the PV and AnV of each sample showed significant differences, and there was a moderate negative correlation between the parameters at *r* = 0.614 (Pearson product-moment correlation coefficient, *p* < 0.05). A high AnV signifies that the decomposition of primary oxidation products is progressing; i.e., the oil in which the samples of barley flour, buckwheat flour, and Job’s tears flour were fried tended to proceed to secondary oxidation compared with the wheat sample. However, AV and PC were low in all sample oils, indicating that the oil deterioration did not progress. The AV was extremely low, and limited free fatty acid was produced during frying. In Europe, the PC is used to regulate the oil used for frying [25]. According to the regulations, frying oil with 25%–27% PC should be changed, and the PC of the frying oil samples in this study were 5.6%–6.0% lower than the specified range in the regulation. We suggest that the oil samples used in this study began to deteriorate; in some of the oil samples, we observed foaming, which indicates deterioration. Nevertheless, as mentioned above, not all the oil samples deteriorated in the frying process.

Deteriorated oil causes oxidative rancidity and taste deterioration, and it contains components that are harmful to the body. These components, which are generated by the deterioration of the oil, are absorbed in deep-fried products with the frying oil and reduce the quality of the oil used for frying as well as the deep-fried products. The toxicity of the deteriorated oil has been studied, and it has been suggested that the cause of toxic effects in heating oil is due to polymerization [26]. In this study, the deterioration level of the oil, including the polymer, was evaluated using PC. However, the PC of the three samples was not higher than that of the wheat flour. It is presumed that the risk of health damage did not increase through the use of these three flours. However, the deterioration of oil in this study was extremely low, and it was not at a level to judge the harmful effects on the human body. Although there was no evident difference in the deterioration between the frying oil samples in the results of this study, it may be necessary to increase the number of frying samples and the heating time to evaluate the deterioration level of each oil sample over time.

Barley, buckwheat, and Job’s tears contain antioxidant components [27,28,29]. Polyphenol was one of the components that was measured in this study. The flours of these samples, especially buckwheat, had much higher polyphenol content than the wheat flour and the amounts thereof ranked in the same order as those for the radical scavenging assay. In addition, the correlation between the radical scavenging activity and polyphenol content was *r*_s_ = 0.953 (Spearman’s rank correlation coefficient, *p* < 0.001), suggesting that polyphenol in those flours is an active antioxidant that contributes to the high antioxidant capacity of tempura, which is fried at a high temperature.

Therefore, the fried samples using the three flours, particularly the buckwheat and Job’s tears flour samples, showed high peroxyl radical scavenging capacity as well as polyphenol content, and the use of barley, buckwheat, and Job’s tears flours for cooking may result in tempura containing a higher antioxidant capacity than that prepared using wheat flour.

**Figure 4:**
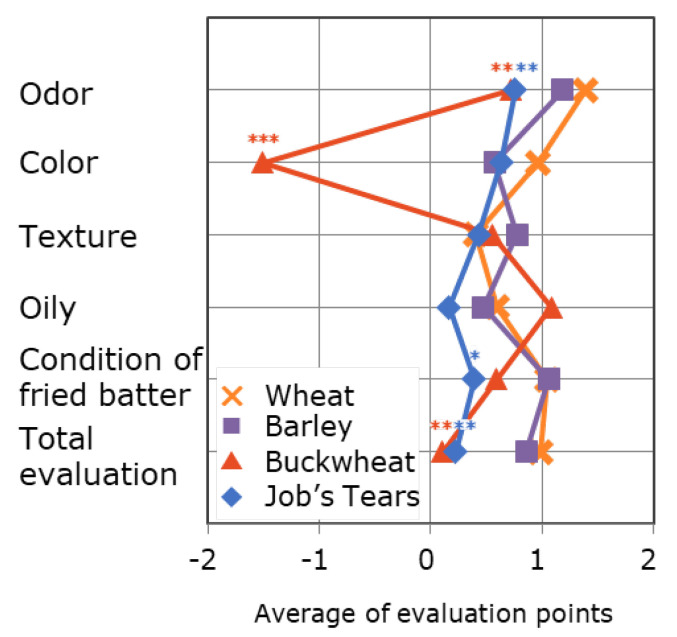
Preference sensory evaluation. Each value is expressed as the mean for each criterion. *** *p* < 0.001, ** *p* < 0.01, * *p* < 0.05 compared with the wheat sample (Dunnett test), *n* = 49. Panel: female university students and teachers aged 18–26 years. Evaluation method: 5-point scoring method of −2 (least preferred) to 2 (preferred). Evaluation sample: sweet potato tempura using each flour examined in this study in the batter. These data were previously reported in [10].

People on dietary restrictions, such as caloric control, tend to avoid tempura because it is a deep-fried product. We previously examined the quality and palatability of tempura using flours of barley, buckwheat, and Job’s tears and found that the odor indices of those samples decreased compared with that of raw batter of the three flours and that oil absorption was approximately the same as or lower than that of the wheat sample. In the preference sensory evaluations, those samples received good evaluation, except for the grayish color of the buckwheat sample (Figure 4) [10]. Although it has already been evaluated that devising a suitable preparation method for tempura is effective in improving its texture and suppressing oil absorption [1,2,3,4], our results suggest that the use of barley, buckwheat, and Job’s tears is a useful substitute for wheat flour when cooking tempura because it maintains its favorable characteristics, such as crispy texture, fragrant smell, and reduction in oil absorption. Moreover, the present study demonstrated that the use of those flours increases the antioxidant capacity of tempura as a new added value. Therefore, the use of barley, buckwheat, and Job’s tears flours is expected to allow more people to enjoy tempura in a healthier manner. The finding that deep-fried products can acquire an antioxidant capacity by substituting wheat flour with barley, buckwheat, and Job’s tears flours is a significant benefit that could be applicable to other deep-fried foods as well.

## Figures and Tables

**Table 1:** Peroxide value (PV) and *p*-anisidin value (AnV) of oil after frying in the four flours examined in this study.

	PV	AnV
Wheat	4.82 ± 1.66 ^a^	39.3 ± 1.98 ^a^
Barley	2.61 ± 1.22 ^b^	53.2 ± 6.58 ^b^
Buckwheat	3.02 ± 1.26 ^b^	43.8 ± 1.30 ^a,b^
Job’s Tears	3.15 ± 1.71 ^b^	45.6 ± 3.00 ^a,b^

The index of oil deterioration of the oil used to fry each of the 48 samples was measured to evaluate primary oxidation. Each value is expressed as the mean ± SD (*n* = 3). Different letters a-b indicate a significant difference (*p* < 0.05, Tukey test).
